# The Trans-facial Approach for Simultaneous Resection and Reconstruction of Retromolar Trigone Tumors: A Pilot Study

**DOI:** 10.1007/s12663-024-02226-0

**Published:** 2024-06-15

**Authors:** Arjun Gurmeet Singh, Manasi Bavaskar, Florida Sharin, Hitesh Singhavi, Rathan Shetty, Poonam Joshi, Sudhir Nair

**Affiliations:** 1 Head and Neck Surgery, ACTREC, Tata Memorial Centre, Homi Bhabha National Institute, Mumbai, Maharashtra India; 2 Head and Neck Surgery, University Hospital of Leicester, Leicester, UK; 3 Head and Neck Surgery, Fortis Hospital, Mumbai, Maharashtra India

**Keywords:** Retromolar trigone lesion, Trans-facial approach, Nasolabial flap

## Abstract

**Introduction:**

Early retromolar trigone (RMT) lesions are difficult to access and free tissue transfer is often an overkill for such small lesions. The aim was to devise a novel surgical approach that would aid the resection without raising a cheek flap and simultaneously provide a local reconstructive option for small lesions in the RMT.

**Methodology:**

This study was to demonstrate the outcomes of the “trans-facial” approach used to simultaneously access and reconstruct small RMT tumors through an islanded nasolabial flap. Patients with histologically proven squamous cell carcinoma of RMT requiring surgery were included from January 2021 to September 2022. Case selection was done based on the location of the disease and its size (cT1/T2). All needed bone and soft tissue resection via per oral trans-facial approach, along with an ipsilateral neck dissection. The technique is described along with their post-operative and pathologic outcomes.

**Results:**

Out of the eight patients included in this study, six underwent a bi-alveolar marginal resection and reconstructed using the trans-facial approach. No major complications were noted in the post-operative period. 50% were pT1 tumors and 75% were pN0 status. One patient had a close margin; while, the others had adequate resection margins. All patients were followed up for a median of 18 months with a locoregionally controlled status.

**Conclusion:**

The trans-facial approach can be a suitable option with a reasonable oncologic outcome to address small RMT lesions.

## Introduction

More than a third of the global oral cancer burden is contributed by South Asia, largely due to the high incidence of tobacco use in the region [1]. Smokeless tobacco use is the highest in the world and leads to the involvement of specific subsites such as the gingivobuccal complex and the retromolar trigone [2]. Involvement of the latter has always been dealt with caution due to the various routes of spread. Moreover, due to its proximity to the muscles of mastication, any treatment, be it radiotherapy or surgery, often can result in severe trismus and debilitation.

Early lesions of the retromolar trigone (RMT) often present without the involvement of the underlying mandible. However, they tend to involve the tonsilo-lingual sulcus as well as part of the adjoining soft palate. Adequate resection of such lesions often comprises marginal mandibulectomy and partial upper alveolectomy along with wide excision of the adjoining mucosa and soft tissues. To gain adequate access to these lesions, a lateral or midline lip split approach is often utilized [3]. This reduces the options for reconstruction using local flaps such as the nasolabial flap. Regional flaps such as the pectoralis major myocutaneous flap (PMMC) is often bulky and can cause significant functional and cosmetic morbidity and may not be suitable for all patients [4]. These defects must be reconstructed with a pliable skin-lined flap like the free radial artery forearm flap. However, in low-resource settings or where a free tissue transfer is not possible, the use of local or pedicled flaps might be useful.

Hence, we attempted a novel technique to approach these early RMT tumors, keeping in mind the associated reconstructive challenges. Here, we demonstrate a “trans-facial” approach for RMT tumors that includes reconstruction using an islanded Nasolabial Flap (NLF).

## Patients and Methods

This study was aimed to demonstrate the outcomes of a novel “trans-facial” approach used to simultaneously access and reconstruct small RMT tumors through an islanded Nasolabial Flap (NLF). Patients with newly diagnosed squamous cell carcinoma of RMT region requiring surgery were included from January 2021 to September 2022. Case selection for this specific approach was done base on the location of the lesion and clinically T1 or T2 status. A total of eight patients were included in this study. The study was approved by the Institutional Ethics Committee (IEC). Radiological assessment was performed to determine skin involvement along with any underlying bone erosion.

Treatment decisions were made as per the disease management group’s joint clinic consisting of surgical, medical, radiation oncologists as well as a radiologist and pathologist. Patients were excluded if other head and neck sites were involved, previously received any treatment or if the disease process warranted a segmental mandibulectomy or skin excision.

**Fig. 1:**
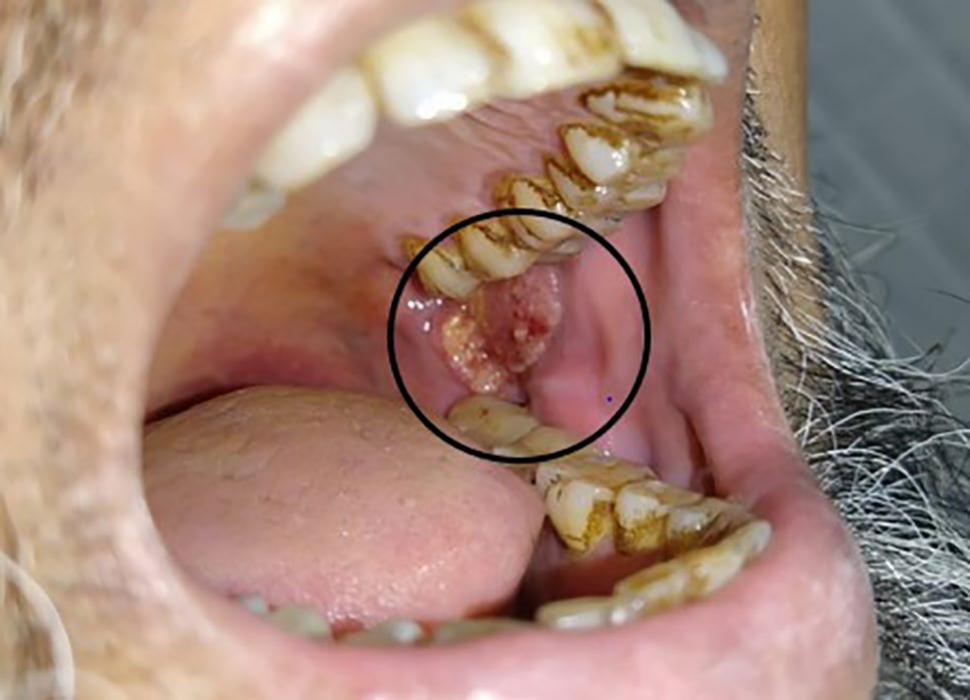
Case selection for Trans-facial approach- Retromolar trigone lesion

Stringent case selection included RMT or posterior buccal mucosa lesions planned for surgical excision of the primary and appropriate for a local or regional flap reconstruction, along with ipsilateral neck dissection was done. A mouth opening of at least 20 mm at presentation was required. A minimum of 30 mm tumor free mucosal margin from the commissure was also needed as shown in Fig. 1, so that the base of the nasolabial flap would not be near the tumor excision area. This was confirmed by examining under anesthesia as well as radiological mapping and estimation of the approximate defect size. All patients underwent surgical resection with a clinical discernable margin of at least 1 cm. This could entail a marginal mandibulectomy with or without partial upper alveolectomy using per oral and trans-facial approach.

### Surgical Procedure

The surgical plan included the completion of the ipsilateral selective neck dissection before commencing with the primary resection. The extent of neck dissection was based on the status of neck metastasis and the level of involvement. For cN0 patients, clearance of ipsilateral levels I to III/IV was performed; while, level V was included in cN + cases with lower neck involvement or clinical extracapsular spread (ECS).

**Fig. 2:**
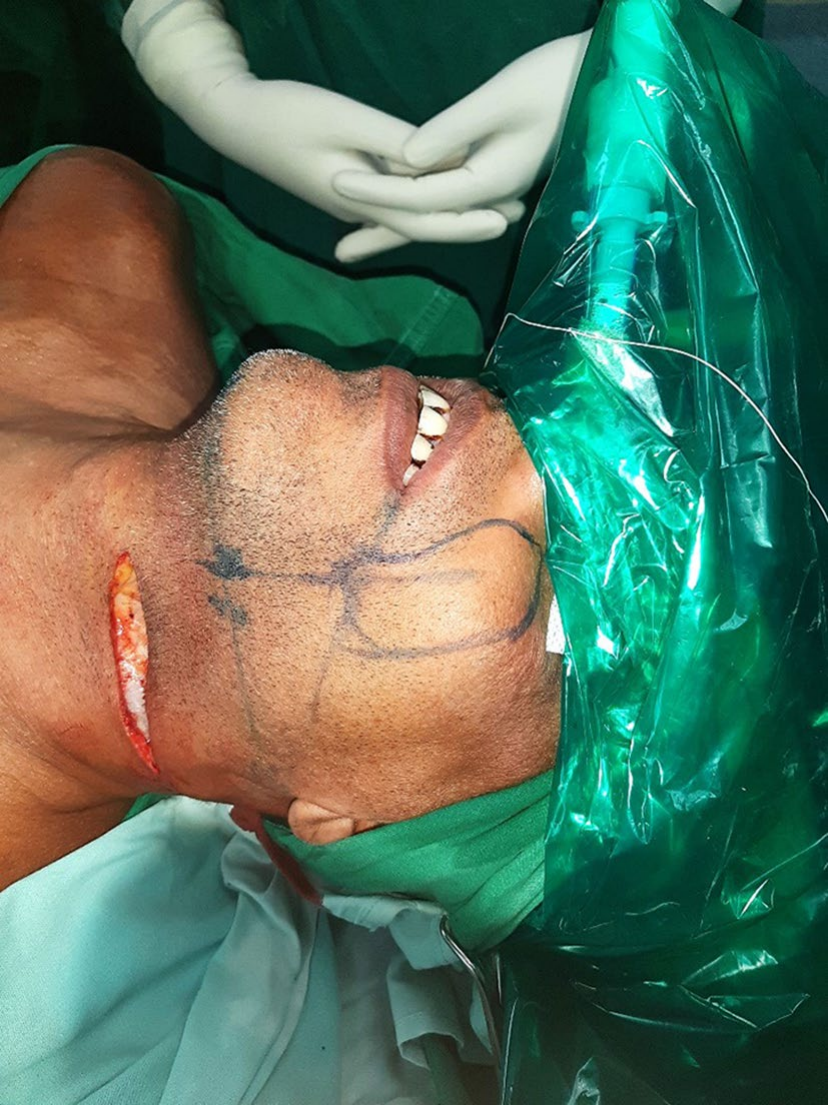
Marking of Islanded Nasolabial Flap

**Fig. 3:**
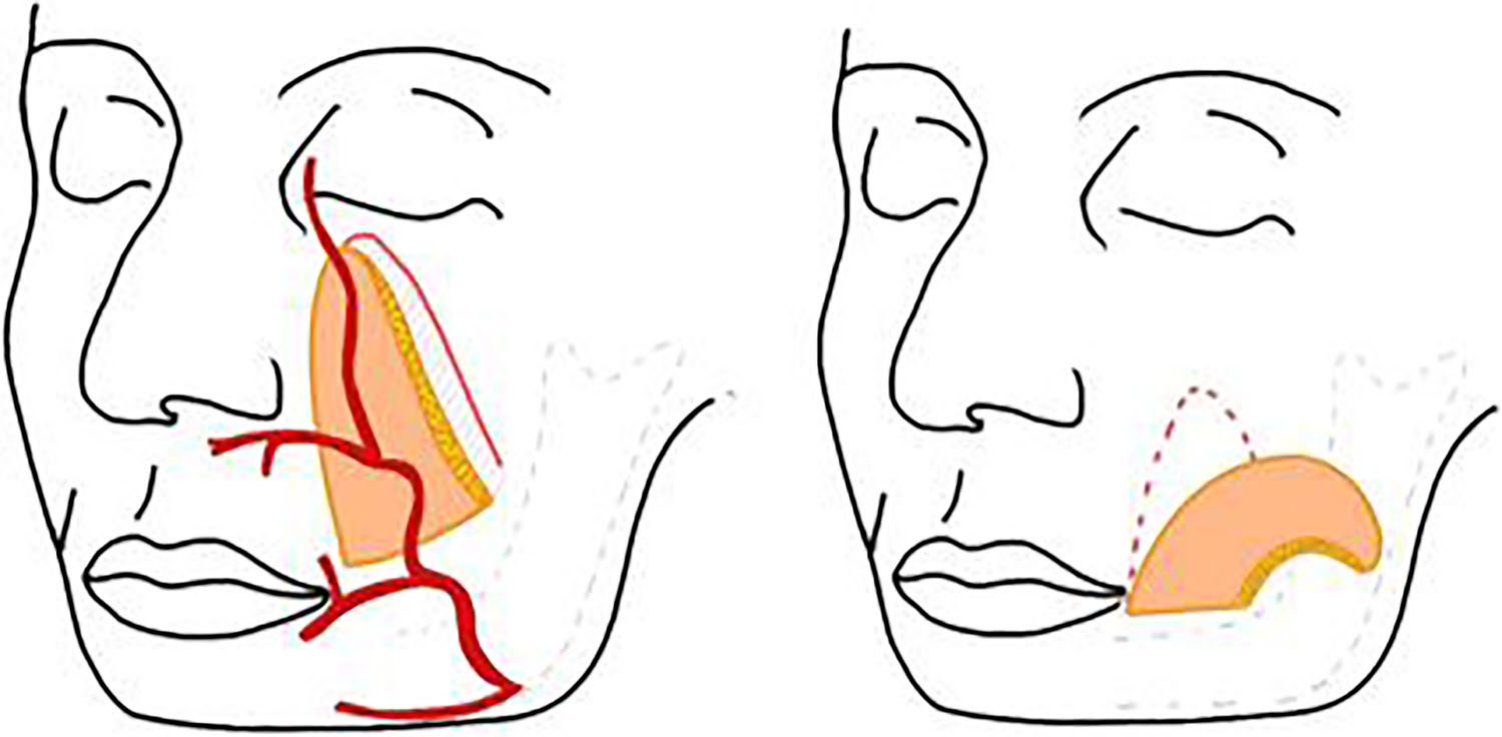
Schematic diagram of NLF based on facial vessels and its reach to RMT lesion

During the neck dissection, care was taken to preserve the facial artery and vein in continuity across the lower border of the mandible. In case the facial vessels were not preserved, the nasolabial flap was not islanded and instead based on a random pattern blood supply with a broad base. After the neck dissection was complete, the planned nasolabial flap was marked (Fig. 2). We started by confirming the surface marking of the facial artery at the lower border of the flap using Mason’s point or a Doppler probe [5]. Once traced, a broad flap is marked based on the defect size. Figure 3 represents a schematic diagram of the Nasolabial flap and its reach to the retromolar trigone region.

The mucosa is incised intraorally, confirming the adequacy of the base of the nasolabial flap. The medial edge of the flap was incised and the facial vessels and its labial branches were identified and preserved by careful dissection. The superior/distal end of the vessel is then ligated and the incision is extended as per the marked flap design. The flap is elevated with the skin, subcutaneous tissue along with the underlying muscle by carefully preserving the artery and veins in the flap. Once the flap is partially raised, we enter the oral cavity by connecting through the anterior mucosal margin.

**Fig. 4:**
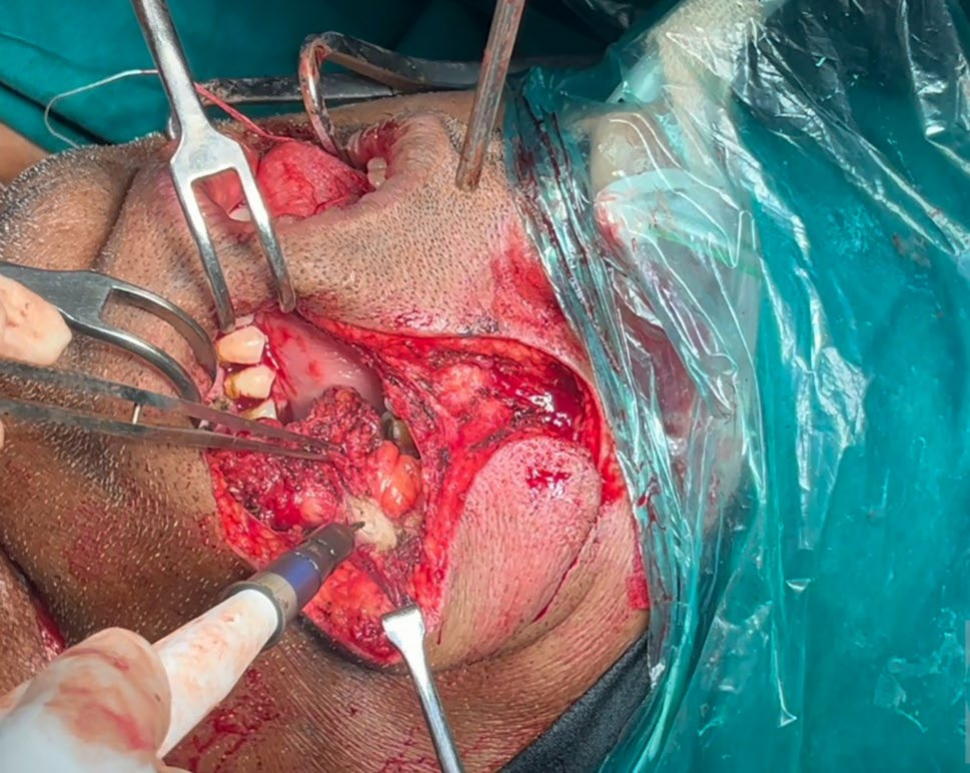
Resection of the tumor through the Trans-facial approach

The anterior mucosal resection margin in the ipsilateral buccal mucosa should correspond to the posterior margin of the modified nasolabial flap. Next, the anterior mucosal cut is deepened to open into nasolabial defect and sufficiently extended to improve access. Further excision of the tumor can be completed through a combined approach using this defect and through the oral cavity (Fig. 4). Due to the wide access obtained transfacially, marginal mandibulectomy with coronoidectomy as well as an upper aleveolectomy can be performed. The specimen is then rotated outward and the posterior tonsillar and soft palate mucosal margins can be accessed intraorally—might be difficult to perform through the defect due to the specimen obstructing vision.

**Fig. 5:**
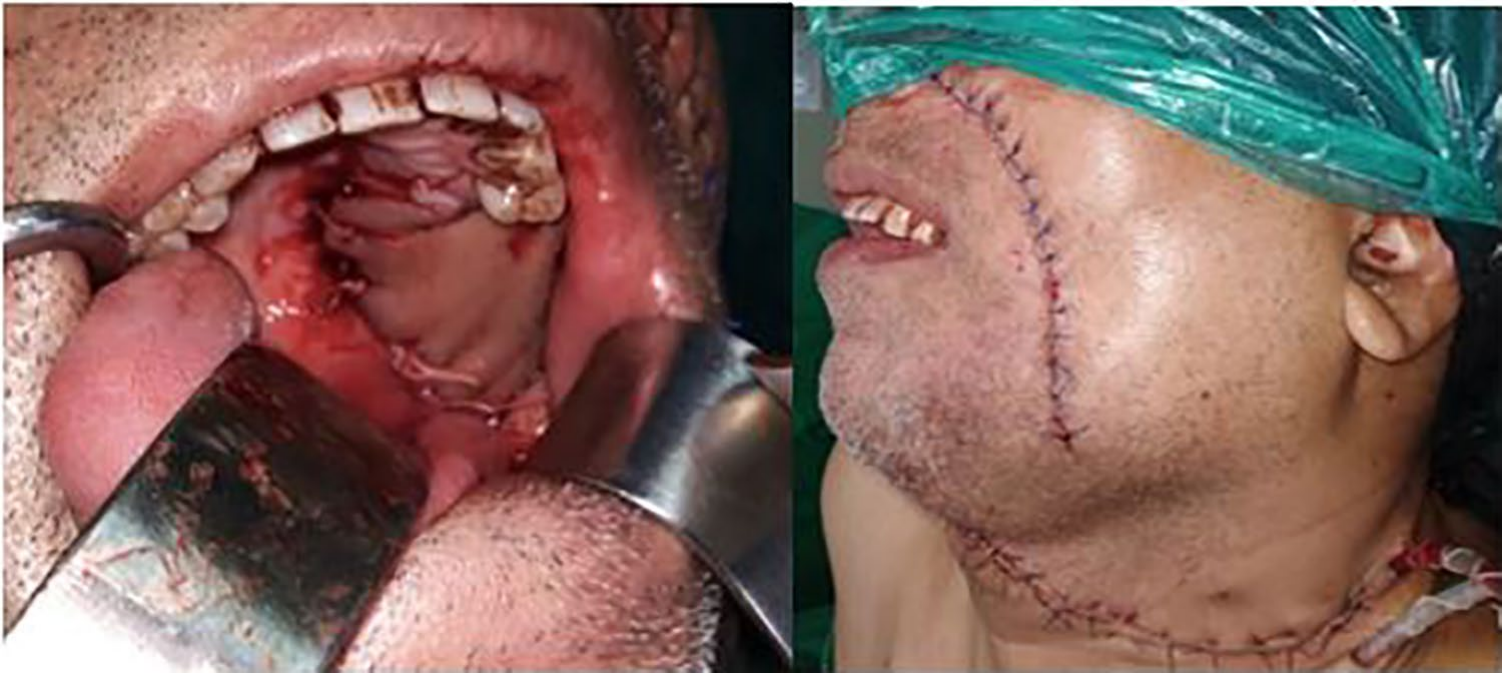
NLF used for reconstruction and final closure

Once the specimen is delivered, the margins are assessed. The nasolabial flap is sutured in the defect (Fig. 5). Once the defects is covered adequately, the donor site is carefully closed along the nasolabial crease avoiding and correcting any Burrow’s triangle formation and any deviation of the oral commissure (Fig. 5).

The histopathology reports were reviewed to record the distance from tumor to mucosal, soft tissue and bone margin, separately, in grossing as well as microscopic examination. Adjuvant radiotherapy (RT) was given when depth of invasion was more than 10 mm, or more than 5 mm with other adverse factors like perineural invasion (PNI) or poor grade of differentiation, or in the presence of positive neck nodes. Concomitant chemotherapy (CCRT) was added in cases of positive margins or presence of extracapsular spread (ECS). All other clinical and pathologic parameters were obtained from the electronic medical records of the hospital.

## Results

All eight patients were included in this study and underwent resection of the primary tumor via the trans-facial approach with a modified nasolabial flap. The demographic details of the patients are compiled in Table 1. All participants were tobacco chewers, male, and between the age group of 35 to 56 years. Clinically and radiologically, they were classified as T1 or T2 tumors with cN0 status. Six patients underwent marginal mandibulectomy and partial upper alveolectomy as part of the resection while 2 underwent only a marginal mandibulectomy. No major intraoperative events were present. The mean operative time was 72 min ± 18 min for the primary tumor resection through the trans-facial approach. An additional 41 min ± 12 min was present if the flap was islanded completely on the facial vessels. All patients had an uneventful post-operative period with a mean hospital stay of 5 days (± 2 days).

**Table 1:** Demographic details

Total number of patients	8
Mean age	45.3 years
Subsite	Buccal mucosa—RMT region
Clinical T stage	
cT1	4
cT2	4
Pathological T stage	
pT1	3
pT2	4
No residual tumor	1
Pathological N stage	
pN0	6
pN +	2
Margin status	
Inadequate (< 5 mm)	1
Adequate (> 5 mm)	7
Marginal mandibulectomy alone	2
Marginal mandibulectomy with upper alveolectomy	6
Adjuvant radiotherapy	4

There were no flap-related complications or oro-cutaneous fistulas in any of the cases. One patient developed a parotid fistula which was addressed with an anticholinergic agent during the hospital stay. In two patients, a controlled fistula was left at the base of the nasolabial defect which was corrected after 4 and 6 weeks, respectively. Half of the patients were pT1 tumors and 75% were pN0 status. One patient had an inadequate margin of 2 mm (superior bone margin) on final histopathology but was free of tumor. The same patient also had a poorly differentiated tumor, lymphovascular emboli and two positive neck nodes for which adjuvant therapy was advised. Another patient had a final diagnosis of hyperplastic squamous mucosa, though the preoperative biopsy was moderately differentiated squamous cell carcinoma. All patients were longitudinally followed up to 18 months (median) with no wound-related complications reported. On follow-up, 88% were alive and locoregionally controlled (*n* = 7) and 12% of the cohort developed recurrence and is alive with disease (*n* = 1).

## Discussion

The aim of performing any oncologic procedure is to offer the patient a reasonable functional, esthetic and oncologic outcome. With the surgical evolutions in the last few decades, balancing these objectives has become a reality by minimizing the access needed to perform the procedure without compromising oncologic outcomes. In this regard, these advances have been led primarily by technology in the form of minimally invasive cameras, lenses, and even robotic systems. These newer surgical techniques reported are also reliant on the development of enabling technology. Hence, very few fundamental techniques in surgery have seen any change. We present our novel technique to access certain oral cavity lesions that maximizes the principles mentioned before.

The most common technique used to access posteriorly based lesions that are not amenable to transoral resection is the elevation of a cheek flap [6]. This leads to an eventual communication between the oral cavity and neck. This communication can lead to major complications such as delayed wound healing, infections, and oro-cutaneous fistula eventually leading to the possibility of secondary hemorrhage. These can lead to a delay in receiving adjuvant therapy, translating into reduced oncologic outcomes.

The trans-facial technique is primarily indicated for posteriorly based lateral oral cavity lesions, such as the retromolar trigone, buccal mucosa, and palatal lesions, where a conventional transoral resection might be difficult. This may be due to restricted mouth opening caused by the disease or severe submucous fibrosis, in which case, the retraction of soft tissue is very difficult. Additionally, the planned reconstruction of these lesions is also a challenge as most free tissue transfers might be larger than the defect. Local and regional flaps have been mentioned in the literature with good outcomes.

The basic philosophy behind the trans-facial approach is to avoid contaminating the sterile neck field with the oral cavity and eventually reduce these inadvertent complications. Since access to the primary site can be achieved through the incision for the nasolabial flap itself, the elevation of a cheek flap is avoided. Splitting the lip is also avoided improving the function of the oral aperture and overall esthetics. Neurologic functions of the marginal mandibular nerve and the mental nerve are also preserved as these are not encountered for access [7].

As with all surgical approaches, a meticulous case selection is required to avoid inadequate margins or poor esthetic and functional outcomes. The possible limitations of this technique include limited applicability in field cancerized mucosa, large dentate segment of bone to be resected which would be difficult to deliver through the trans-facial defect, and in situations where the nasolabial flap would not be possible or indicated. While this novel technique has worked in our setup, its applicability in other centers and surgeons is needed.
